# Supplementary material for: Prime-Boost Vaccination Based on Nanospheres and MVA Encoding the Nucleoprotein of Crimean-Congo Hemorrhagic Fever Virus Elicits Broad Immune Responses
Source: Vaccines (Basel). 2025 Mar 10;13(3):291. doi: 10.3390/vaccines13030291 (PMC11946443; doi:10.3390/vaccines13030291)
Supplement: Supplementary file 1 [file vaccines-13-00291-s001.zip › Figure S1. Gating strategy used to analyze specific T-cell responses.pdf]

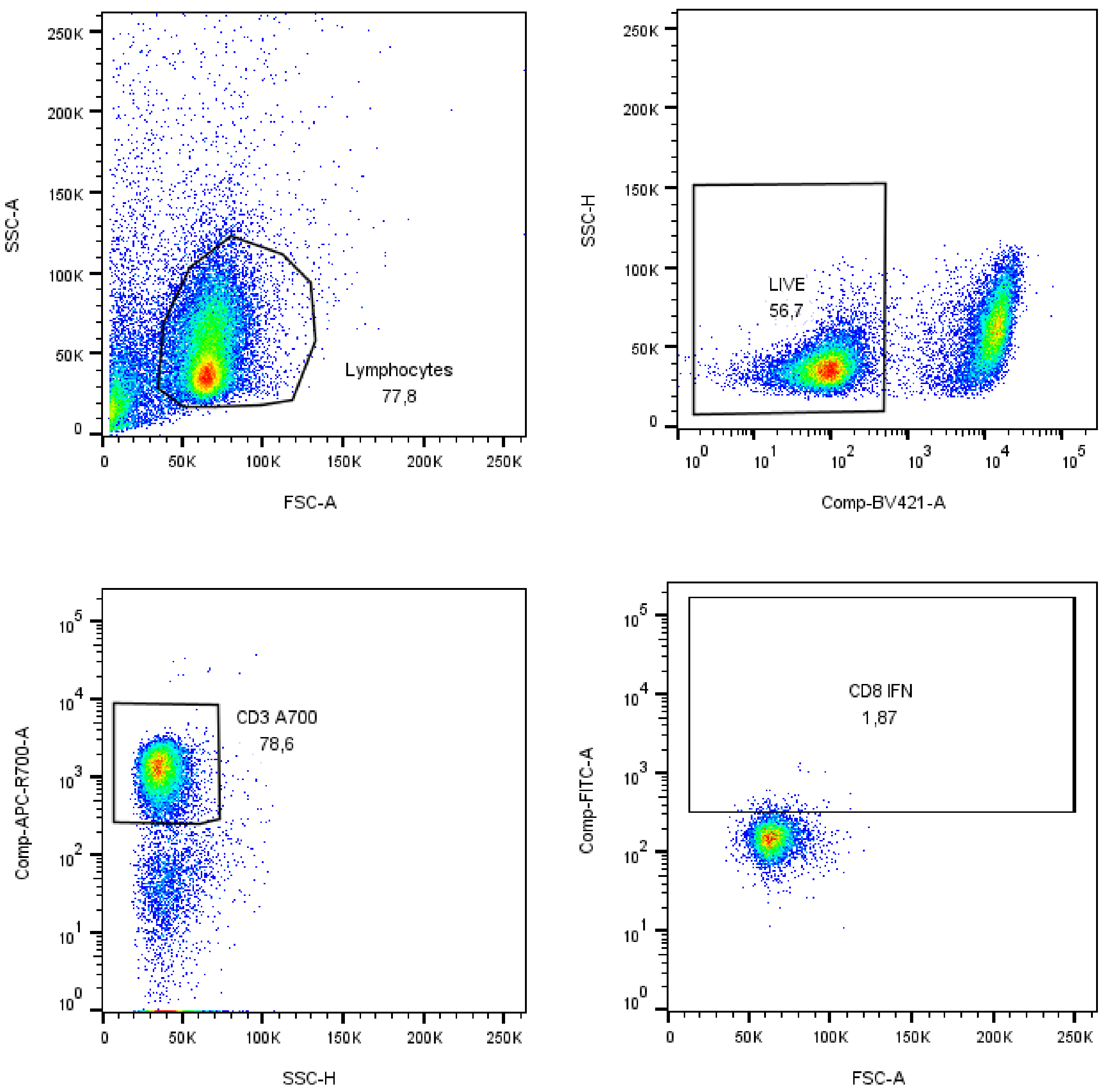

Supplementary figure 1. Gating strategy used to analyze specific T-cell responses. Lymphocytes were gated by SSC vs FCS density plot. Live lymphocytes were selected by negative zombie staining and T cells were determined as CD3+ positive cells. CD8+ T cells were analyzed for expression of IFN-γ.
