# Supplementary material for: Prime-Boost Vaccination Based on Nanospheres and MVA Encoding the Nucleoprotein of Crimean-Congo Hemorrhagic Fever Virus Elicits Broad Immune Responses
Source: Vaccines (Basel). 2025 Mar 10;13(3):291. doi: 10.3390/vaccines13030291 (PMC11946443; doi:10.3390/vaccines13030291)
Supplement: Supplementary file 1 [file vaccines-13-00291-s001.zip › File S1. The original Western blot figures.pptx]

## Slide 1
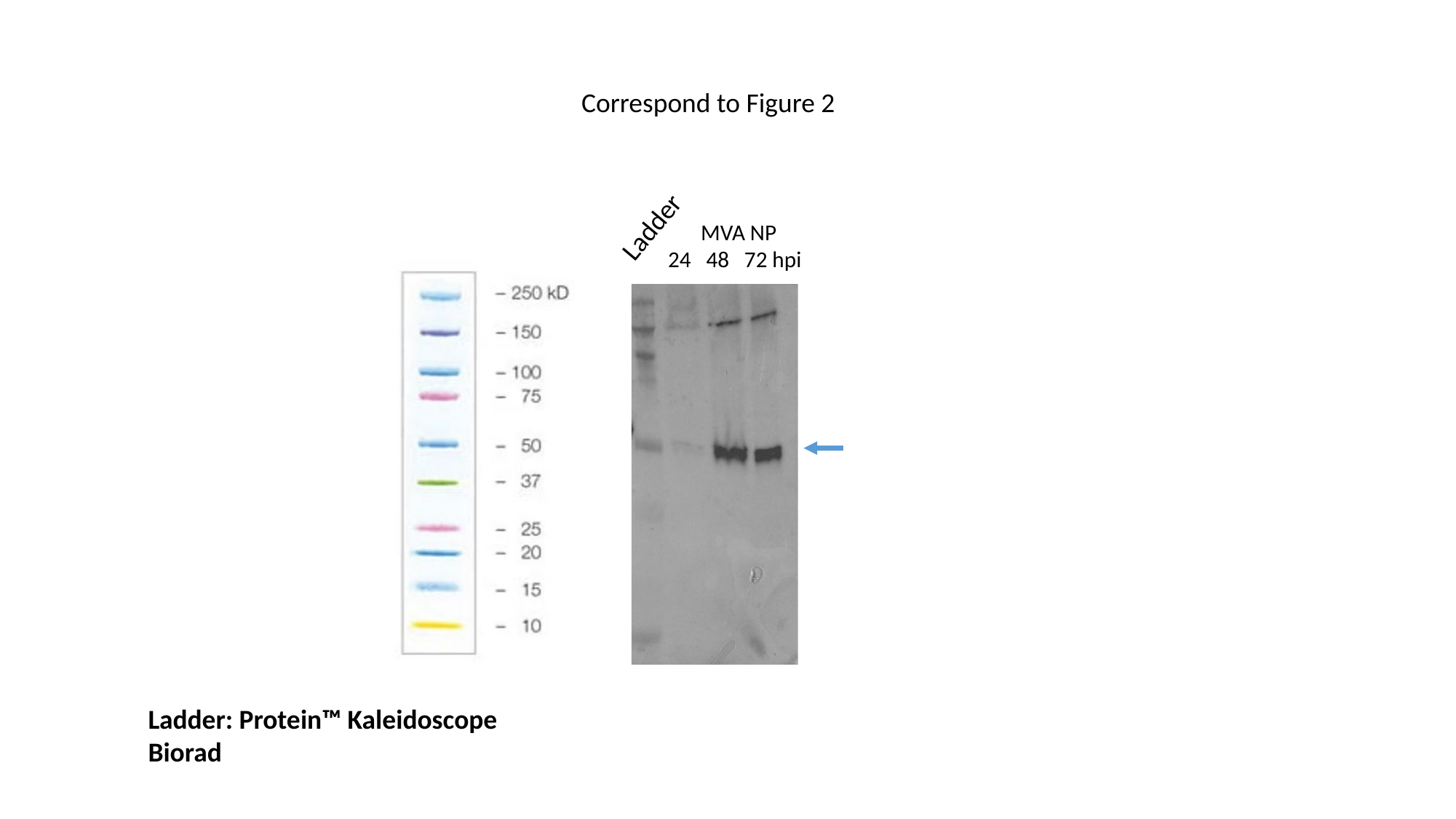

Correspond to Figure 2
Ladder
	MVA NP
 24 48 72 hpi
Ladder: Protein™ Kaleidoscope
Biorad

## Slide 2
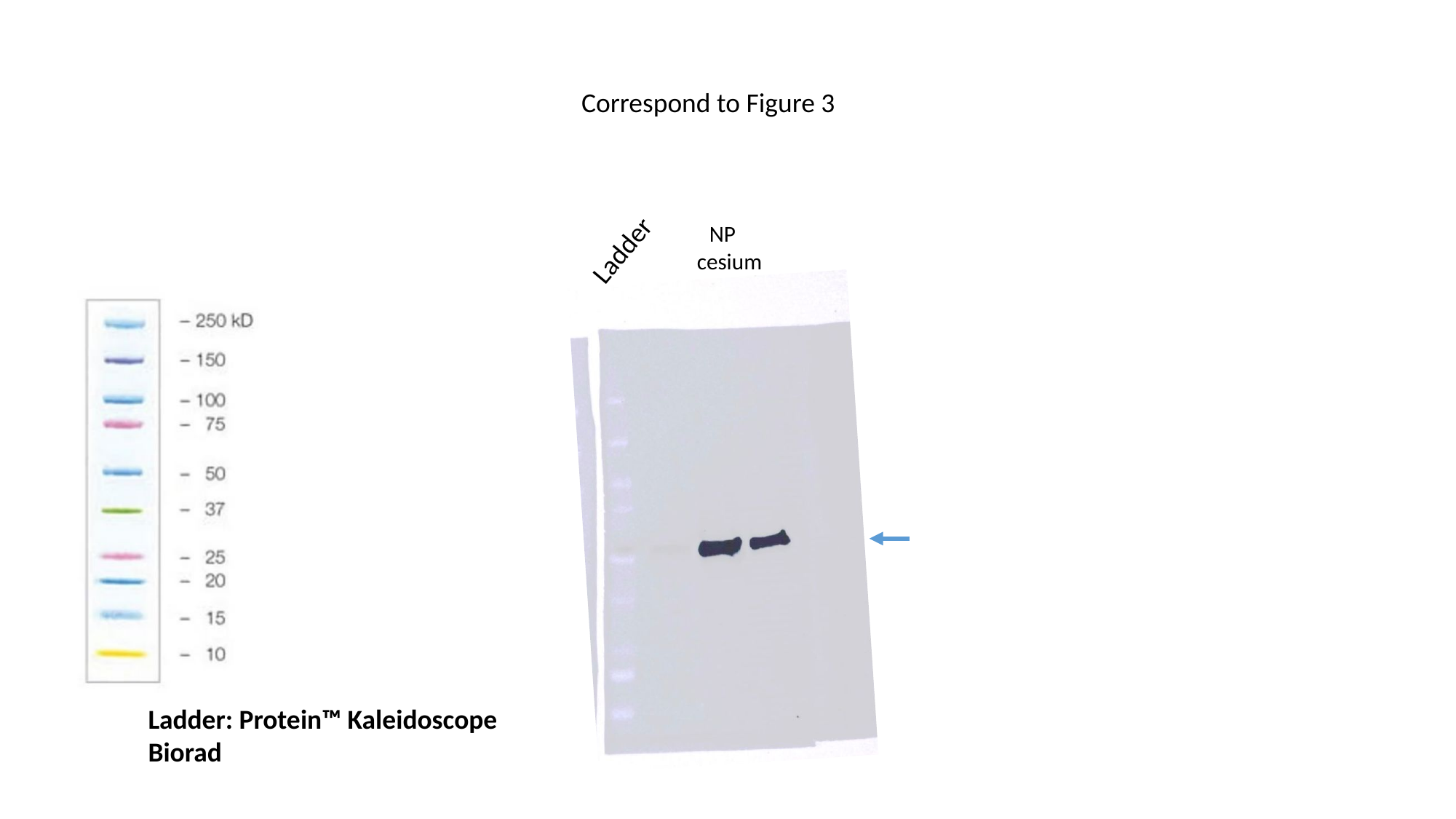

Correspond to Figure 3
	NP
 cesium
Ladder
Ladder: Protein™ Kaleidoscope
Biorad

## Slide 3
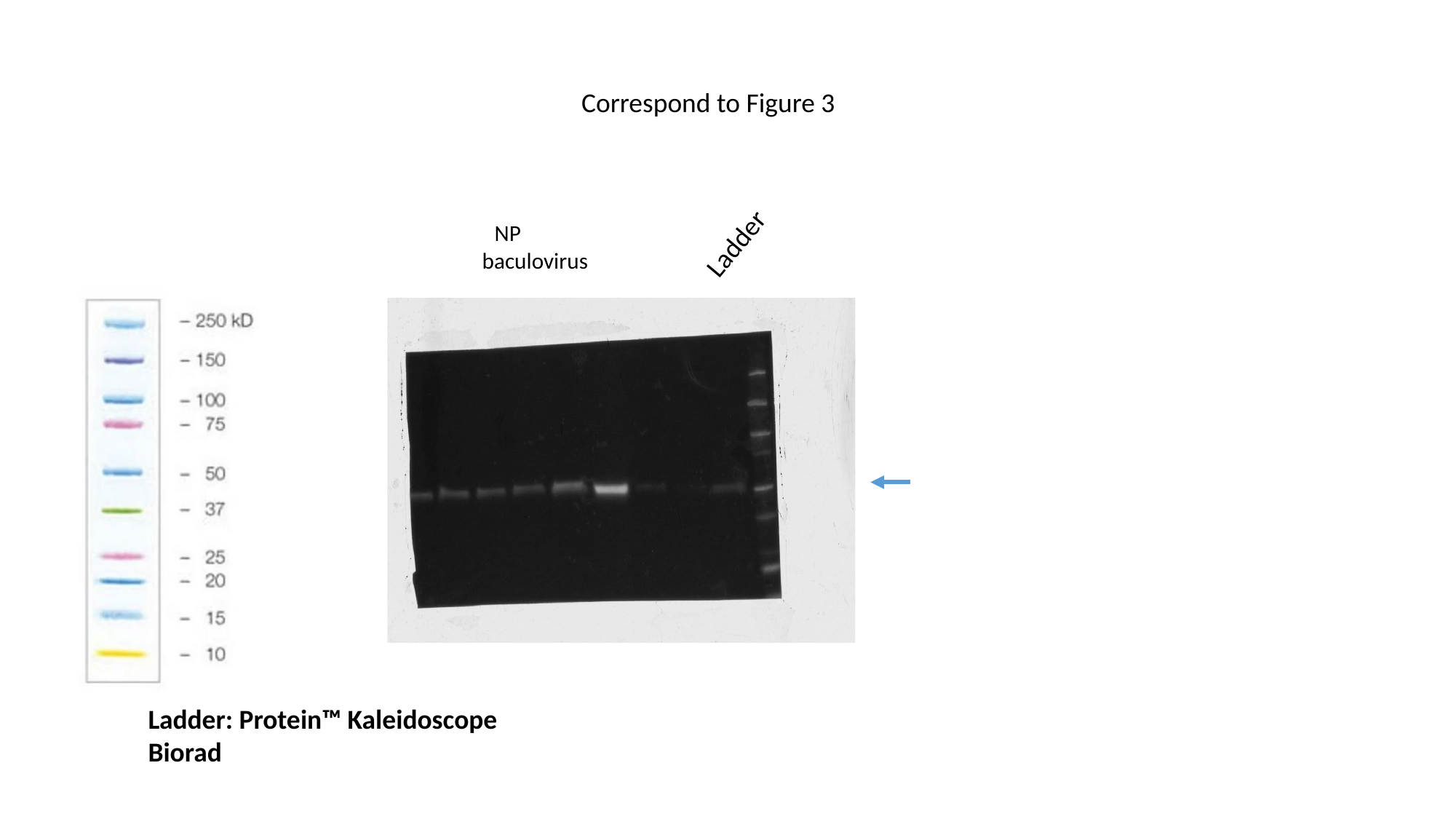

Correspond to Figure 3
	NP
 baculovirus
Ladder
Ladder: Protein™ Kaleidoscope
Biorad
